# Comparing bound entanglement of bell diagonal pairs of qutrits and ququarts

**DOI:** 10.1038/s41598-023-29211-w

**Published:** 2023-02-04

**Authors:** Christopher Popp, Beatrix C. Hiesmayr

**Affiliations:** Faculty of Physics, University of Vienna, Währingerstrasse 17, 1090 Vienna, Austria

**Keywords:** Quantum physics, Quantum information

## Abstract

We compare the classification as entangled or separable of Bell diagonal bipartite qudits with positive partial transposition (PPT) and their properties for different dimensions. For dimension $d \ge 3$, a form of entanglement exists that is hard to detect and called bound entanglement due to the fact that such entangled states cannot be used for entanglement distillation. Up to this date, no efficient solution is known to differentiate bound entangled from separable states. We address and compare this problem named separability problem for a family of bipartite Bell diagonal qudits with special algebraic and geometric structures and applications in quantum information processing tasks in different dimensions. Extending analytical and numerical methods and results for Bell diagonal qutrits ($d=3$), we successfully classify more than $75\%$ of representative Bell diagonal PPT states for $d=4$. Via those representative states we are able to estimate the volumes of separable and bound entangled states among PPT ququarts ($d=4$). We find that at least $75.7\%$ of all PPT states are separable, $1.7\%$ bound entangled and for $22.6\%$ it remains unclear whether they are separable or bound entangled. Comparing the structure of bound entangled states and their detectors, we find considerable differences in the detection capabilities for different dimensions and relate those to differences of the Euclidean geometry for qutrits ($d=3$) and ququarts ($d=4$). Finally, using a detailed visual analysis of the set of separable and bound entangled Bell diagonal states in both dimensions, qualitative observations are made that allow to better distinguish bound entangled from separable states.

## Introduction

Quantum technology leverages quantum phenomena for better performance than classical methods for applications like computing, communication, simulation, metrology and cryptography^1–5^. Quantum information theory provides the theoretical formalism for processing tasks using quantum mechanical systems^6^. One of the characteristic properties of a quantum system that allows realizing information processing with superior performance compared to classical systems is entanglement. Besides its relevance for our general understanding of nature and the interpretation of quantum theory^7–9^ it provides one of the main resources to realize applications in various fields ranging from quantum teleportation to medical applications for the detection of cancer cells^10–13^. The simplest system to observe entanglement is the bipartite system of two two-level quantum systems, called qubits. Currently, most applications are based on these quantum systems of dimension $d=2$, but recently, interest in higher dimensional systems like “qutrits” for $d=3$, “ququarts” for $d=4$ or “qudits” for general *d* is growing due to potential advantages and new observable phenomena^14,15^. Bell states^16^ are special sets of entangled states which can be used as basis for the corresponding Hilbert space. They are highly relevant for applications due to the fact that they are maximally entangled states. Originally introduced for $d=2$, they can be generalized for higher dimensions^17–19^.

In this paper we analyze mixtures of maximally entangled bipartite Bell states, with focus on $d=3$ and $d=4$. Those states are locally maximally mixed, meaning that there is no correlation in the respective subsystems. Depending on the mixing probabilities of the $d^2$ pure Bell basis states, a general mixed state can be entangled or not, in which case it is separable. The Peres-Horodecki criterion, also known as PPT (positive partial transposition) criterion^20,21^, provides an efficient method to detect a state as entangled, if the partially transposed density matrix of a given quantum state has at least one negative eigenvalue, in which case the state is called “NPT”. Otherwise it is called “PPT”. For $d=2$, all entangled states are NPT, but for $d \ge 3$, also PPT entangled states exist^22^. While NPT entangled states can be “distilled”^22^ to result in fewer strongly entangled states, this process is not possible for PPT entangled ones. For this reason PPT entanglement is also called “bound” entanglement, which has been extensively investigated since its discovery by the Horodecki family, e.g. in Refs.^23–33^. 2014, it has also been observed in experiment, using photons entangled in their orbital angular momentum^34^. Many applications like teleportation or superdense coding^6^ require strongly entangled states for reliable performance. However, if a given state is transformed to a bound entangled one, this resource is bound for immediate application, since it cannot be used to distill strongly entangled states. For this reason, it is important to know about structure of bound entanglement in a given system and to be able to detect those states reliably, so that operations that result in binding the resource entanglement in certain states can be avoided. However, the “separability problem” to differentiate separable and PPT entangled states has been proved to be NP-hard^35,36^ in general and lacks an efficient solution if the dimension of the system is not small. Existing methods to detect PPT entangled states^27–33^ are often strongly limited in the number of states they can detect and are not efficient in higher dimensions. Likewise, no efficient method to decide whether a PPT state is separable or bound entangled states is known for Bell diagonal qudits, which are known to be highly relevant for practical applications^37^. However, special families of these states have strong symmetries that can be leveraged for the analytical and numerical analysis of its properties regarding the entanglement structure^19,34,38^. In particular, the analytical structure of mixed Bell states generated by Weyl-Heisenberg transformations^18^ allows to derive several criteria to detect separability and entanglement^19,38^. Furthermore, an efficient geometric representation of the states, symmetries and entanglement witnesses^39,40^ makes the system well applicable for numerical methods.

Recently, analytical and numerical methods were combined to solve the separability problem for the system in three dimensions in an “almost complete” way^41^. Given any unknown PPT state, the developed methods allow the classification of this state as separable or bound entangled with a probability of success of $95 \%$. Moreover the classification allows the determination of the relative volumes of separable, PPT and NPT entangled states. It was further shown, that a significant share of the PPT states of bipartite, Bell diagonal qutrits are bound entangled (at least $13.9\%$), making this system exceptionally well suited to study this exotic form of entanglement regarding its detection, use in information processing tasks and implications for nature. It is expected that the dimension of the system has a large influence on the structure and the relative shares of entanglement classes, which is a focus of this contribution. While approximations of the relative volumes of separable states in general systems in dependence on the dimension exist^42,43^, the precise numbers depend on the specific system and are not known.

The aim of this work is to extend and apply those methods, used to successfully characterize the system for qutrits^41^, for $d=4$, to draw conclusions about the structure of entangled and separable states as well as the effectiveness of their detectors and to compare the results to $d=2$ and $d=3$. The paper is organized as follows: First, the system to be analyzed is defined and relevant methods to generate states and to investigate its entanglement structure are presented for general dimension. Second, we analyze the set of PPT states for $d=4$. We quantify the share of this set in the total system and the relative volumes of separable and (bound) entangled states within and compare to other dimensions. Then, the applied criteria to detect separability and entanglement are compared for their effectiveness in different dimensions. Finally, we leverage the special properties of the system to visualize the set of separable and bound entangled states for $d=3$ and $d=4$. The visual analysis demonstrates relations between the algebraic structure of Bell diagonal mixtures and geometric restrictions on the set of separable and bound entangled states. This might be leveraged for the detection of bound entanglement and separability.

## Methods

Consider the Hilbert space ${\mathscr {H}} = {\mathscr {H}}_1 \otimes {\mathscr {H}}_2$ for the bipartite system of two qudits of dimension *d*. In this work we analyze mixtures of maximally entangled orthonormal Bell states $|{\Omega _{k,l}}\rangle \in {\mathscr {H}}$ with $k, l = 0, 1, \ldots , (d-1)$ generated by applying the Weyl operators^18^ $W_{k,l}$ to one qudit of the shared maximally entangled state $|{\Omega _{00}}\rangle \equiv \frac{1}{\sqrt{d}} \sum _{i = 0}^{d-1} |{ii}\rangle$:

1 $$\begin{aligned} |{\Omega _{k,l}}\rangle \equiv W_{k,l} \otimes {\mathbb {1}}_d |{\Omega _{00}}\rangle \end{aligned}$$

where $W_{k,l} \equiv \sum _{j=0}^{d-1}w^{j \cdot k} |{j}\rangle \langle {j+l \pmod d}|,~w = e^{\frac{2 \pi i}{d}}$. Mixing the density matrices, or “Bell projectors”, $P_{kl} \equiv |{\Omega _{k,l}}\rangle \langle {\Omega _{k,l}}|$ with mixing probability $c_{k,l}$ defines Bell diagonal states with respect to the above defined Weyl operators and the system of interest for this work:

2 $$\begin{aligned} {\mathscr {M}}_d \equiv \lbrace \rho = \sum _{k,l = 0}^{d-1}c_{k,l} P_{k,l}~ |~ \sum _{k,l = 0}^{d-1}c_{k,l} = 1, c_{k,l} \ge 0 \rbrace \end{aligned}$$

By taking the partial trace with respect to one of the subsystems, the reduced state of any state in ${\mathscr {M}}_d$ is maximally mixed, so all information is in the correlation of the combined state and not in the subsystems themselves. States with this property are called “locally maximally mixed”. Any state of ${\mathscr {M}}_d$ is equivalent to a point in $d^2$ dimensional Euclidean space by identifying the mixing probabilities $c_{k,l}$ with coordinates in real space. Due to the normalization of the $c_{k,l}$, the set of these points forms a standard simplex. Referring to the “magic Bell basis” of Wootters and Hill^44^ ${\mathscr {M}}_d$ is also known as “magic simplex”^19,38,45^.

The properties of the Weyl operators $W_{k,l}$ imply a linear ring structure or “discrete phase space” for operators indexed by the tuples (*k*, *l*) based on succeeding application of these operators^19^. This can be seen via the Weyl relations^18^ (addition defined modulo *d*):

3 $$\begin{aligned} W_{k_1,l_1}W_{k_2,l_2}=& \,w^{l_1 k_2}~W_{k_1+k_2, l_1+l_2} \end{aligned}$$

4 $$\begin{aligned} W_{k,l}^\dagger=& \,w^{k l}~W_{-k, -l} = W_{k,l}^{-1} \end{aligned}$$

**Figure 1:**
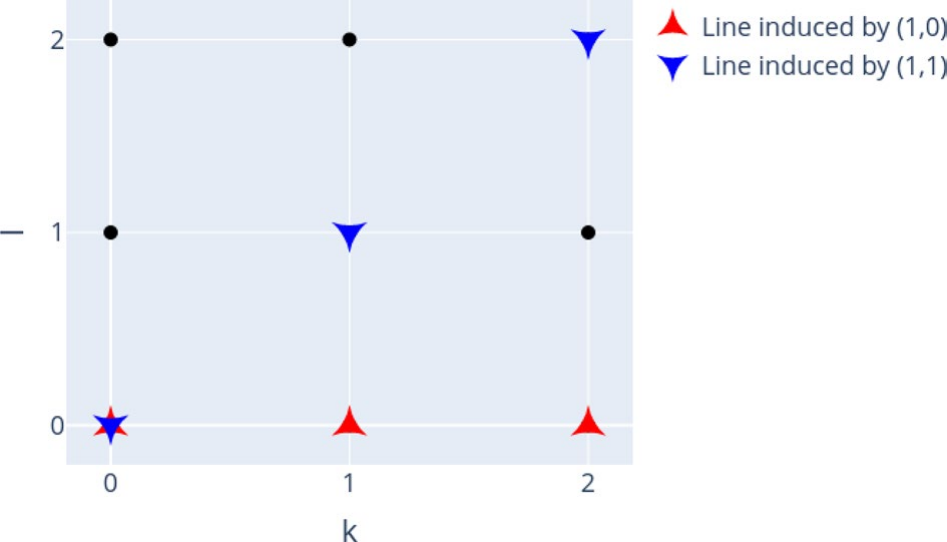
Phase space and exemplary induced subgroups called “lines” for $d=3$. Figure created with Ref.^46^.

**Figure 2:**
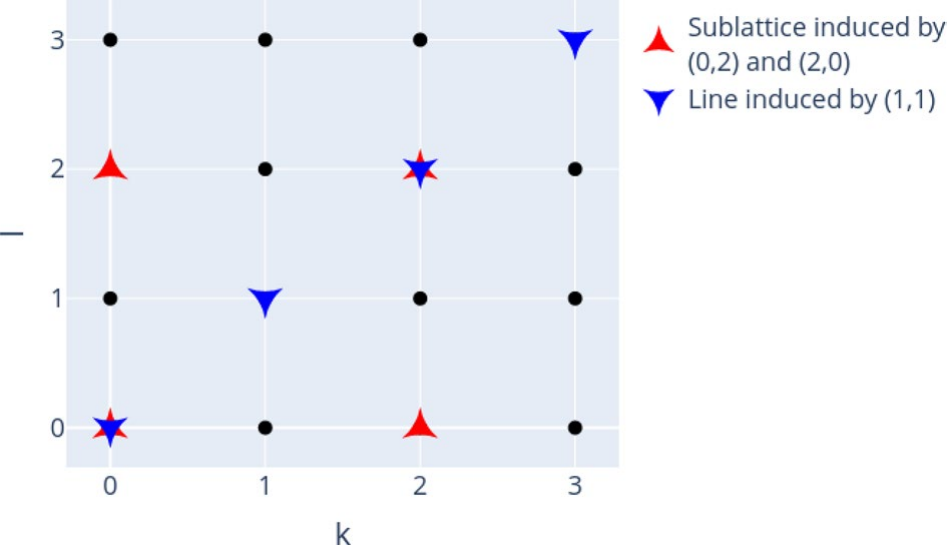
Phase space and exemplary induced subgroups called “lines” and “sublattices” for $d=4$. Figure created with Ref.^46^.

In Figs. 1 and 2 we visualize this phase space as lattice of $d \times d$ vertices, each vertex corresponding to the Weyl operator (and thus also to a Bell state via eq.(1)) with according indices (*k*, *l*). Depending on the dimension *d*, several subgroups exist and for this work subgroups containing *d* elements are of special relevancy, as they can be related to the structure of the sets of separable and bound entangled states. In general, a subgroup is defined by its generating elements. In case *d* is prime, all subgroups of *d* elements are generated by one of the Weyl operators. Highlighting the vertices (here red and blue) corresponding to a subgroup generated by $W_{k,l}$ induces “lines” in the discrete phase space (see Fig. 1). This is different for non-prime dimensions, where subgroups of *d* elements can additionally be generated by two Weyl operators whose indices contain proper divisors of *d* in which case “sublattices” are formed in the phase space (see e.g. Fig. 2). For more details, consult Ref.^19^. We can also use Figs. 1 and 2 to specify “subgroup states” by assigning each highlighted subgroup to a state which consists of equal mixtures of all corresponding Bell states.

### Relevant subsets

The properties of ${\mathscr {M}}_d$ allow the definition of special subsets related to the entanglement properties of contained states, which have been investigated with respect to separability and (bound) entanglement^45,47^.

#### Enclosure polytope

The enclosure polytope is a superset of all states with positive partial transposition. It was shown^19^ that all states that have at least one mixing probability $c_{k,l}$ exceeding 1/*d* are necessarily entangled and can be detected by the Peres-Horodecki criterion (PPT criterion). As NPT entangled states they can be distilled by local operation and classical communication (LOCC), and are therefore called “free” and not “bound” entangled^22^. The enclosure polytope is defined as:

5 $$\begin{aligned} {\mathscr {E}}_d \equiv \lbrace \rho = \sum _{k,l = 0}^{d-1}c_{k,l} P_{k,l}~ |~ \sum _{k,l = 0}^{d-1}c_{k,l} = 1, c_{k,l} \in [0, \frac{1}{d}] \rbrace \end{aligned}$$

Using the representation of ${\mathscr {M}}_d$ in Euclidean space, ${\mathscr {E}}_d$ forms a bounded polytope.

#### Kernel polytope

For each of the subgroups of *d* elements induced by the Weyl operators (indexed by $\alpha$), a special “subgroup” or “sublattice state” $\rho _{\alpha }$ can be defined, which is known to be a separable state^19^. In general, these subgroup states are equal mixtures of *d* Bell states corresponding to a subgroup with probability 1/*d*, e.g. the line state $\rho _{\alpha _1} = \frac{1}{d} \sum _{k=0}^{d-1} P_{k,0}$ (see also Figs. 1 and 2). This gives rise to a kernel polytope ${\mathscr {K}}_d$, which is defined as convex mixture of these separable line or sublattice states with *d* elements:

6 $$\begin{aligned} {\mathscr {K}}_d \equiv \lbrace \rho = \sum _\alpha \lambda _{\alpha } \rho _{\alpha }~ |~ \lambda _\alpha \ge 0, \sum _\alpha \lambda _\alpha = 1 \rbrace \end{aligned}$$

All states in the kernel polytope are convex combinations of separable states, consequently each state in ${\mathscr {K}}_d$ is by construction separable and the center corresponds to the maximally mixed state.

### Bell diagonal state generation in arbitrary dimension

Using the geometric representation of ${\mathscr {M}}_d$ in Euclidean space, any state can be generated by specifying its $d^2$ coordinates $c_{k,l}$. Random sampling as well as deterministic procedures can be used to generate states. It was shown^41^ that via random sampling, uniformly distributed states in ${\mathscr {M}}_d$ can be generated and used to estimate the relative volumes of the entanglement classes of separable, bound entangled and free entangled states in ${\mathscr {M}}_3$. The same method can be used for $d>3$ to generate states in ${\mathscr {M}}_d$ and ${\mathscr {E}}_d$ by drawing the first $d^2-1$ coordinates from a uniform distribution in the range [0, 1] for ${\mathscr {M}}_d$ or [0, 1/*d*] for ${\mathscr {E}}_d$ respectively. The remaining coordinate is then chosen according to the normalization condition. If this is not possible with a non-negative probability, the coordinates do not represent a normalized physical state and is therefore rejected. This form of rejection sampling becomes less effective with growing dimension *d* because the probability of a random state being rejected increases rapidly for $d \ge 5$. However, several methods exist to sample uniformly distributed points on the standard simplex and thus in ${\mathscr {M}}_d$ in any dimension (see Ref.^48^ and the references therein), but these methods are not accessible for sampling of states located only in the polytopes ${\mathscr {E}}_d$ or ${\mathscr {K}}_d$.

### Symmetries and their generation

The ring structure of the Weyl operators $W_{k,l}$ can be used to define linear symmetry transformations^19,38^ acting on states in ${\mathscr {M}}_d$. These transformations act as permutations on the Bell basis projectors $P_{k,l}$ or equivalently as permutations of the coordinates $c_{kl}$ of a state in ${\mathscr {M}}_d$. These symmetry transformations form a group and are known to conserve both the PPT property and entanglement. Together these properties imply the conservation of the entanglement class^41^, meaning that the subsets of separable, bound entangled and free entangled states are mapped to themselves. For numerical implementations as well as for understanding their action on a given state, any linear symmetry *s* can be characterized by their action on the basis projectors $s: P_{k,l}\rightarrow P_{k', l'}$. All elements of this symmetry group can be generated by combined application of the following group generators (all mathematical operations on the indices are defined as$\pmod d$):

- Momentum inversion: $$m: P_{k,l}\rightarrow P_{-k,l}$$
- Quarter rotation: $$r: P_{k,l}\rightarrow P_{k,-l}$$
- Vertical sheer: $$v: P_{k,l}\rightarrow P_{k+l,l}$$
- Translation: $$t_{p,q}: P_{k,l}\rightarrow P_{k+p,l+q}$$ for $$p, q \in (0, \ldots , d-1)$$

Note that the translation of a Bell diagonal state can also be realized by applying a corresponding Weyl operator to the mixed state. This is not the case for the other generators, as their action on each Bell state that contributes to the mixture depends on that state. Due to the finite number of elements (*k*, *l*) in the phase space induced by the Weyl operators $W_{k,l}$, the number of distinct symmetries generated by the generators above is finite as well and can be generated numerically. The number of existing symmetries grows quickly with the dimension. For $d=2$, 24 distinct symmetries can be generated, for $d=3$, 432 and for $d=4$ already 1536 symmetries of this group exist. Taking these symmetries into account is essential for the numerical methods to be effective in the $d^2$ dimensional space.

### Criteria for the detection of entanglement and separability

The separability problem to decide whether a given mixed quantum state is separable or entangled has been shown to be NP-hard with respect to the dimension *d* as complexity measure^35,36^ and lacks an efficient general solution by polynomial in time algorithms, which is also the case for states in ${\mathscr {M}}_d$ for general dimension *d*. It is currently unclear, whether an efficient general solution exists for the separability problem in ${\mathscr {M}}_d$. For $d=2$, however, all entangled states can be detected by the PPT criterion^20,21^ and recently, an almost complete solution was presented^41^ for $d=3$, in the sense that any random unknown PPT state in ${\mathscr {M}}_3$ can be classified numerically with a probability of success of $95\%$. The methods used in this work can be equivalently used or extended to be applicable for $d>3$. In the following we introduce those methods shortly. For more detailed information, the reader is referred to Ref.^41^ and the references therein.

#### E1: PPT criterion

The “Positive Partial Transpose (PPT)” or “Peres-Horodecki” criterion^20^ detects entanglement for a bipartite state if it has at least one negative eigenvalue (in which case it is said to be “NPT”). For $d=2$ it detects all entangled states, but for $d \ge 3$ it is only sufficient due to the existence of PPT- or bound entangled states. The partial transpose $\Gamma$ acts on the basis states of a bipartite state as $(|{i}\rangle \langle {j}|\otimes |{k}\rangle \langle {l}|)^{\Gamma } \equiv |{i}\rangle \langle {j}|\otimes |{l}\rangle \langle {k}|$.

#### E2: Realignment criterion

The realignment operation *R* is defined as $(|{i}\rangle \langle {j}|\otimes |{k}\rangle \langle {l}|)_R \equiv |{i}\rangle \langle {k}|\otimes |{j}\rangle \langle {l}|$. The realignment criterion^49^ states that if the sum of singular values of the realigned state $\sigma _R$ are larger than 1, then $\sigma$ is entangled. Like the PPT criterion it is only sufficient for entanglement. Bound entangled states can be detected by this criterion, but it does not detect all NPT states in general.

#### E3: Quasi-pure concurrence criterion

The quasi-pure approximation^47^ $C_{qp}$ of the concurrence^50^ allows the efficient detection of entanglement including its bound form. The approximation takes an explicit form for states in ${\mathscr {M}}_d$: A state $\rho = \sum _{k,l = 0}^{d-1}c_{k,l}P_{k,l} \in {\mathscr {M}}_d$ is entangled if $C_{qp}(\rho ) = \max (0, S_{nm} - \sum _{(k,l) \ne (n,m)} S_{k,l})>0$ where the $S_{k,l}$ are explicitly given by^47^

7 $$\begin{aligned} S_{k,l} = \sqrt{\frac{d}{2(d-1)} c_{k,l} [(1-\frac{2}{d}) c_{n,m} \delta _{k,n} \delta _{l,m} + \frac{1}{d^2} c_{(2n-k)mod~d,(2m-l)mod~d}]} \end{aligned}$$

and (*n*, *m*) is a multi-index of the coordinate of the largest value $\lbrace c_{k,l} \rbrace$.

#### E4: MUB criterion

A set of orthonormal bases $\lbrace B_k \rbrace$ and $B_k = \lbrace |{i_k}\rangle ~|~ i = 0,\ldots , (d-1) \rbrace$ is called “mutually unbiased bases (MUB)” if $\forall k \ne l$: $|\langle {i_k}\rangle {j_l}|^2 = \frac{1}{d} ~~~ \forall i,j = 0, \ldots , (d-1)$. At most $d+1$ MUBs exist^51,52^, in which case it was shown^34,53,54^ that the sum of “mutual predictabilities” obeys

8 $$\begin{aligned} I_{d+1}(\rho _s) = \sum _{k=1}^{d+1} C_k(\rho _s) \le 2 \end{aligned}$$

for all separable states $(\rho _s)$, when defining

9 $$\begin{aligned} C_1(\rho ) = \sum _{i=0}^{d-1} \langle {i_1}|\otimes \langle {(i_1+s)^*}| \rho |{i_1}\rangle \otimes |{(i_1+s)^*}\rangle , \end{aligned}$$

10 $$\begin{aligned} C_k(\rho ) = \sum _{i=0}^{d-1} \langle {i_k}|\otimes \langle {i_k^*}| \rho |{i_k}\rangle \otimes |{i_k^*}\rangle ,~ k=2,\dots ,d+1. \end{aligned}$$

Here, $s = 0,1, \dots , (d-1)$ and $i_k^*$ denotes the complex conjugate vector element. The MUB criterion thus indicates that if any state violates (8), it is entangled. If $s > 0$ and $s \ne d/2$ the MUB criterion allows the detection of PPT entangled states^53^, which was also experimentally demonstrated for entangled photons^34^ in the case $d=3$. Note, that other, inequivalent MUBs exist, including extendible or unextendible sets of bases that contain less than $d+1$ elements^55^. The set of entangled states that are detected by the MUB criterion generally depends on the used MUB. For this work, we use MUBs of $d+1$ elements as given in the Appendix A1 and set $s=2$ for $d=3$ and $s=3$ for $d=4$.

#### E5: Numerically generated entanglement witnesses

An entanglement witness^39^ (“EW”) *W* is an observable which implies an upper bound *U* and also a lower bound^40^ *L* ($U,L \in {\mathbb {R}}$), for separable states $\rho _s$:

11 $$\begin{aligned} L \le \textrm{tr}[\rho _s W] \le U \end{aligned}$$

A state $\rho$ is “detected by W” to be entangled, if $\textrm{tr}[\rho W] \notin [L,U]$. For the system ${\mathscr {M}}_d$, EWs of the form $W = \sum _{k,l = 0}^{d-1}\kappa _{k,l} P_{k,l}$ with $\kappa _{k,l} \in [-1,1]$ can detect all entangled states^19^. In this case $\rho = \sum _{k,l = 0}^{d-1}c_{k,l}P_{k,l}\in {\mathscr {M}}_d$ and $\textrm{tr}[\rho W]= \sum _{k,l = 0}^{d-1}c_{k,l} \kappa _{k,l} \equiv c \cdot \kappa$ using the standard scalar product of the $d^2$-dimensional vectors *c* and $\kappa$ with coefficients $c_{k,l}$ and $\kappa _{k,l}$. Using the geometric representation of ${\mathscr {M}}_d$, an EW defines two $(d^2-1)$-dimensional hyper-planes via $c_L \cdot \kappa = L$ and $c_U \cdot \kappa = U$ and induced halfspaces. Any point in the simplex but outside of the intersection of these halfspaces is entangled. An parameterization of unitaries^56^ can be used to numerically determine the bounds for any EW defined by its coefficients $\kappa _{k,l}$ to create EWs for states in ${\mathscr {M}}_d$ numerically.

Leveraging the geometric characterization of ${\mathscr {M}}_d$, also sufficient criteria to detect separable states have been developed and used to analyze ${\mathscr {M}}_3$^41^. They can be applied for $d>3$ as well and are shortly stated here:

#### S1: Extended kernel criterion

The convexity of the set of separable states can be used to check if an unknown state is contained in the separable hull of known separable states via linear programming. For this work, the implementation of Ref.^57^, was used to check an unknown state for separability based on the convex hull of a given set of separable states. Using known separable states in ${\mathscr {M}}_d$ as vertices, they form a polytope which approximates the convex set of all separable states in ${\mathscr {M}}_d$. The effectiveness of this criterion depends on the quality of this approximation. More separable vertices for the polytope to improve the approximation increase the probability to detect new separable states, but on the other hand the complexity of the linear program also increases. It is therefore important to use vertices that are spatially uniformly distributed and as close to the surface of the set of separable states as possible. The sublattice states $\rho _\alpha$ of the kernel polytope ${\mathscr {K}}_d$ meet those requirements^19^ and by using the entanglement-class-conserving symmetries, more vertices to extend the separable kernel can be generated.

#### S2: Weyl/Spin representation criterion

Based on the Weyl relations^18^

12 $$\begin{aligned} W_{k_1,l_1}W_{k_2,l_2}=& \,w^{l_1 k_2}W_{k_1+k_2, l_1+l_2} \end{aligned}$$

13 $$\begin{aligned} W_{k,l}^\dagger=& \,w^{k l}W_{-k, -l}=W_{k,l}^{-1} \end{aligned}$$

one can see that the Weyl operators form an orthogonal basis in the space of $d \times d$ matrices with respect to the trace norm $\langle A,B \rangle \equiv \textrm{tr}[A^\dagger B]$. Representing a density matrix $\sigma$ as $\sigma = \frac{1}{d} \sum _{k,l = 0}^{d-1}s_{k,l} W_{k,l}$ defines the coefficients of this “Weyl representation” as $s_{k,l} = \textrm{tr}[W_{k,l}^\dagger \sigma ]$. For bipartite states, $W_{\mu , \nu } \equiv W_{\mu _1, \nu _1} \otimes W_{\mu _2, \nu _2}$ the coefficients are indexed as $s_{\mu , \nu }$. It was shown^58^ that if $\sum _{\mu , \nu } |s_{\mu , \nu }| \le 2$, then $\rho$ is separable. This criterion for separability is named “Weyl” or “spin representation criterion”.

### Symmetry classification

The group of entanglement class conserving symmetries (see “Symmetries and their generation”) provide further methods to determine the entanglement class of a given unknown state in ${\mathscr {M}}_d$. First,the set all symmetric states, i.e. the orbit of the unknown state, is generated by application of the according transformation for all generated symmetries. Then, this set is analyzed with respect to the available criteria. If the entanglement class is determined for one of the symmetric states, then all symmetric states are certainly of the same class. This method can additionally be used to generate more states of a certain class for further investigations.

## Results

### Volume of PPT states

A first application leveraging the presented methods is to determine the relative volume of states with positive partial transposition in ${\mathscr {M}}_d$. It was shown^42,43^ that the volume of general separable and bound entangled quantum states decreases exponentially with the dimension of the system. Here, we determine the relative volumes for Bell diagonal states.

**Figure 3:**
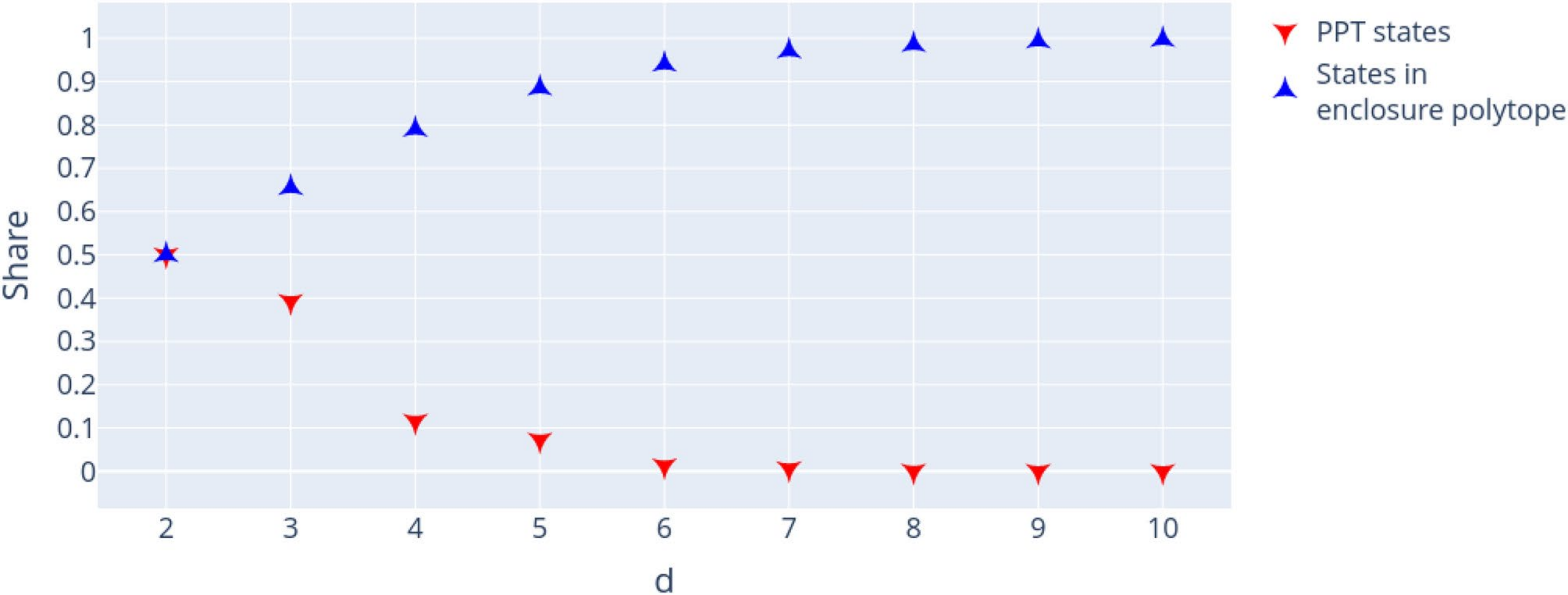
Relative volumes of the enclosure polytope ${\mathscr {E}}_d$ and PPT states in ${\mathscr {M}}_d$ for different dimensions *d*. Figure created with Ref.^46^.

As described in section “Relevant subsets”, all states with positive partial transposition are necessarily located in the enclosure polytope ${\mathscr {E}}_d$ (5) when represented in Euclidean space. This property of ${\mathscr {M}}_d$ yields an upper bound of the relative share of PPT states in the simplex by comparing the total volume of ${\mathscr {M}}_d$ to the volume of ${\mathscr {E}}_d$. The enclosure polytope generally contains both PPT and NPT states, but the ratio depends on the dimension *d*. For $d=2$, all states of ${\mathscr {E}}_2$ are known to be separable and thus PPT^59^, no PPT/bound entangled states exist. For $d=3$ it was numerically shown^41^ that approximately $60.0\%$ of the states in ${\mathscr {E}}_3$ ($39\%$ of all states in ${\mathscr {M}}_3$) are PPT. In order to determine the relative volumes of PPT, we generate a large number of uniformly distributed states for dimensions $d=2, \dots , 10$ and check if they are in the enclosure polytope and if they are PPT. The results are summarized in Fig. 3.

The analysis demonstrates that despite of the fact that the relative volume of the enclosure polytope grows with increasing dimension, the relative number of PPT states quickly decreases. For $d=4$, $11.6\%$ are PPT and the enclosure polytope ${\mathscr {E}}_4$ makes up $79.0\%$ of ${\mathscr {M}}_4$. For $d=5$, the relative PPT volume reduces to $7.3\%$ and already for $d=6$, less than $1\%$ are PPT, although $97.1\%$ of states in ${\mathscr {M}}_6$ are located in ${\mathscr {E}}_6$.

### Entanglement classification of PPT states in ${\mathscr {E}}_d$

In order to compare the entanglement properties of mixed Bell diagonal states for the dimension $d=2,3,4$, we use uniformly generated random states in ${\mathscr {E}}_d$ and classify them with the criteria for separability and entanglement presented above in order to estimate the share of each entanglement class. As mentioned in section “Volume of PPT states”, the classification of bipartite qubits can be completely characterized by the PPT criterion (E1): all states in ${\mathscr {E}}_2$ are PPT and separable, no PPT entangled states exist. For $d=3$, we take the results of the previous investigation^41^, in which $96.1 \%$ of generated states in ${\mathscr {E}}_3$ have been successfully classified. To determine the relative volumes of entanglement classes among the PPT states for $d=4$ with comparable precision, 40000 random states are generated in ${\mathscr {E}}_4$ out of which $96.7\%$ can be successfully classified.

The $60 \%$ of states in ${\mathscr {E}}_3$ and $14.6 \%$ of ${\mathscr {E}}_4$ that have positive partial transposition are labeled as “SEP” or “BOUND” if they are detected as separable or entangled by the criteria E2–E5 or S1–S2. If none of the criteria allows classification, the state is labeled “PPT-UNKNOWN”. Table 1 summarizes the results:

**Table 1:** Relative volumes of entanglement classes among the PPT states for $d=2,3,4$.

Entanglement class	Share of PPT for $d=2$ (%)	Share of PPT for $d=3$ (%)	Share of PPT for $d=4$ (%)
SEP	$100$	$81.0$	$75.7$
BOUND	$0$	$13.9$	$1.7$
PPT-UNKNOWN	$0$	$5.1$	$22.6$

Comparing the numerical classifications for $d=3$ and $d=4$, three noteworthy differences can be seen: First, the relative number of PPT-UNKNOWN states is significantly higher for $d=4$ ($22.6\%$) than for $d=3$ ($5.1\%$), although the success rates ($96.7\%$ and $96.1 \%$) for the classification with respect to the total number of generated states in ${\mathscr {E}}_4$ and ${\mathscr {E}}_3$ are similar. Second, the share of separable states in $d=4$ ($75.7\%$) among the PPT states is quite high, in spite of the large number of yet to be classified PPT states. Third, the number of detected bound entangled states in $d=4$ ($1.7 \%$) is significantly lower than for $d=3$ ($13.9 \%$). Although it is possible that a large part of the PPT-UNKNOWN states are in fact BOUND and thus could potentially be detected by criterion E5, this shows that the detection capability of the analytical criteria (E2–E4) is more limited for $d=4$ than for $d=3$.

### Detection capabilities and relations of applied criteria

The detection and thus differentiation between bound entangled and separable states is the core of the separability problem. Hence, the detection capabilities of the presented detectors for the classes SEP and BOUND are of special interest. Table 2 shows for each relevant criterion and dimension *d* the share of detected states among all SEP, respectively BOUND, classified states.

**Table 2:** BOUND and SEP detectors and their detection shares for $d=3$ and $d=4$.

Entanglement class	Criterion	Share in class for $d=3$ (%)	Share in class for $d=4$ (%)
SEP	S1	$100$	$100$
SEP	S2	$1.7$	$0$
BOUND	E2	$74.9$	$74.7$
BOUND	E3	$19.1$	$2.2$
BOUND	E4	$13.5$	$0$
BOUND	E5	$86.6$	$68.7$

On the one hand, one notices that the strongest detectors for bound entangled Bell diagonal qutrits are also the most successful detectors in $d=4$, namely E2 and E5. Relative to the total amount of detected bound entangled states, E2 seems to perform equally well in both dimensions. However, due to the large amount of PPT-UNKNOWN states, the relative detection power could be much worse. Still, E2 is clearly the strongest applied analytical criterion for $d=3$ and $d=4$. The criterion based on combining many numerically generated witnesses, E5, detects a large share of the identified BOUND states for both analyzed dimensions, however, the share is lower for $d=4$ ($86.6 \%$ for $d=3$, $68.7 \%$ for $d=4$). Considering the PPT-UNKNOWN states, the true detection capability of this criterion might be even below the determined share of $68.7 \%$ for $d=4$, even though more EWs were used for $d=4$ (approximately 22700 compared to 16700 for $d=3$). This indicates that a single randomly generated numerical EW most likely is a weaker detector for the higher dimension.

On the other hand, the other detectors are clearly weaker in $d=4$ compared to $d=3$. The second strongest criterion in $d=3$, E3, detects $19.1 \%$ of the BOUND qutrits in, while only $2.2 \%$ in $d=4$. Again, the true detection capability is likely even below that due to the large number of unclassified PPT states. E4 detects a significant share ($13.5 \%$) of bound entangled qutrits while no PPT entangled qudits for $d=4$. Likewise, S2 detects no states as separable for $d=4$.

**Figure 4:**
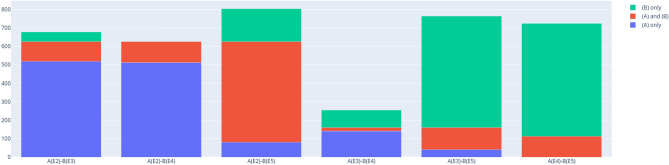
Pairwise comparison of number of exclusively (blue and green) and jointly (red) detected states for $d=3$. Figure created with Ref.^46^.

**Figure 5:**
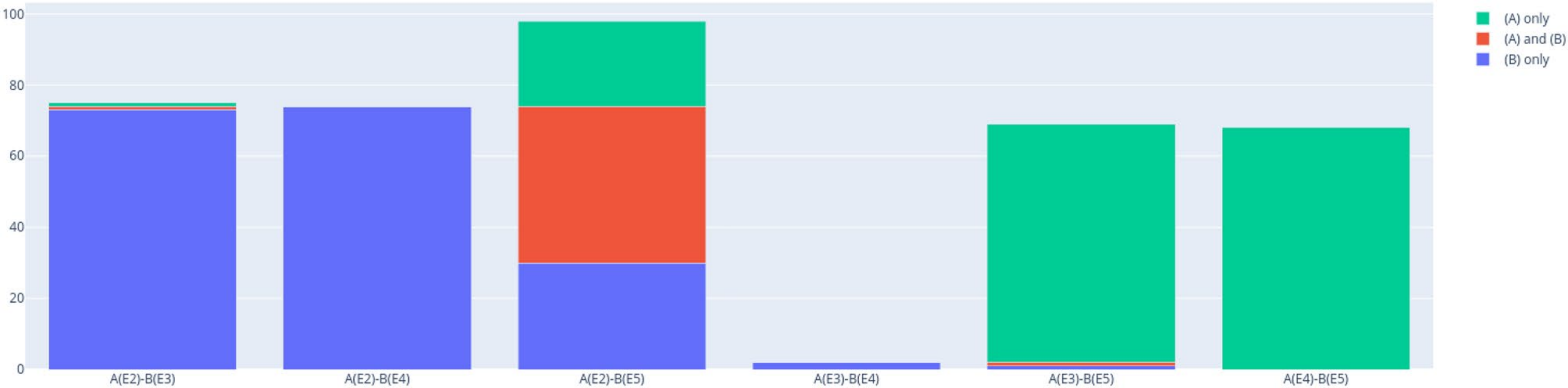
Pairwise comparison of number of exclusively (blue and green) and jointly (red) detected states $d=4$. Figure created with Ref.^46^.

These differences are also clearly reflected when comparing the criteria E2–E5 pairwise as shown in Figs. 4 and 5. The only criteria that have a significant number of jointly detected states in $d=4$ are the detectors E2 and E5 ($44.4 \%$ of combined detected states), although the share is smaller than for $d=3$ ($67.7 \%$), confirming the reduced effectiveness of numerical EWs in the higher dimension. For $d=4$, the other pairs are rather trivial, because of the very low number of detected states by E3 and E4. It should be noted, however that E3 detects one bound state that is neither detected by E2 nor by E5. Interestingly, this criterion also detects significant shares of bound entanglement that are not detected jointly by E2 or E5 in $d=3$.

A final remark can be made related to the purity $\textrm{tr}\rho ^2$ of detected bound entangled states $\rho$. For $d=3$, the least pure states were detected by the criterion E3 but not E2 or E4. The few detected states for $d=4$ do not allow to confirm this observation, although it can be noted that the least pure bound state is also uniquely detected by E3.

### Visual analysis of separable and bound entangled states in ${\mathscr {M}}_3$ and ${\mathscr {M}}_4$

**Figure 6:**
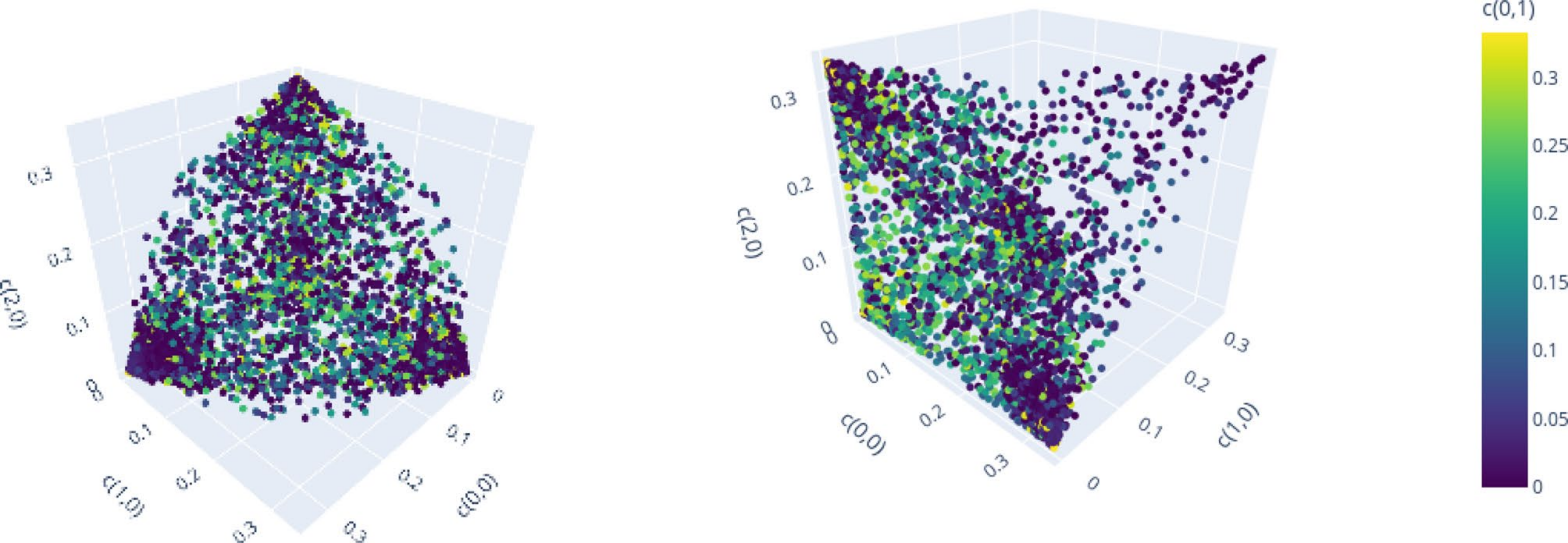
$(c_{0,0}, c_{1,0}, c_{2,0}, c_{0,1})$ for optimized separable states for $d=3$, demonstrating strong correlations of on-line coordinates. Figure created with Ref.^46^.

**Figure 7:**
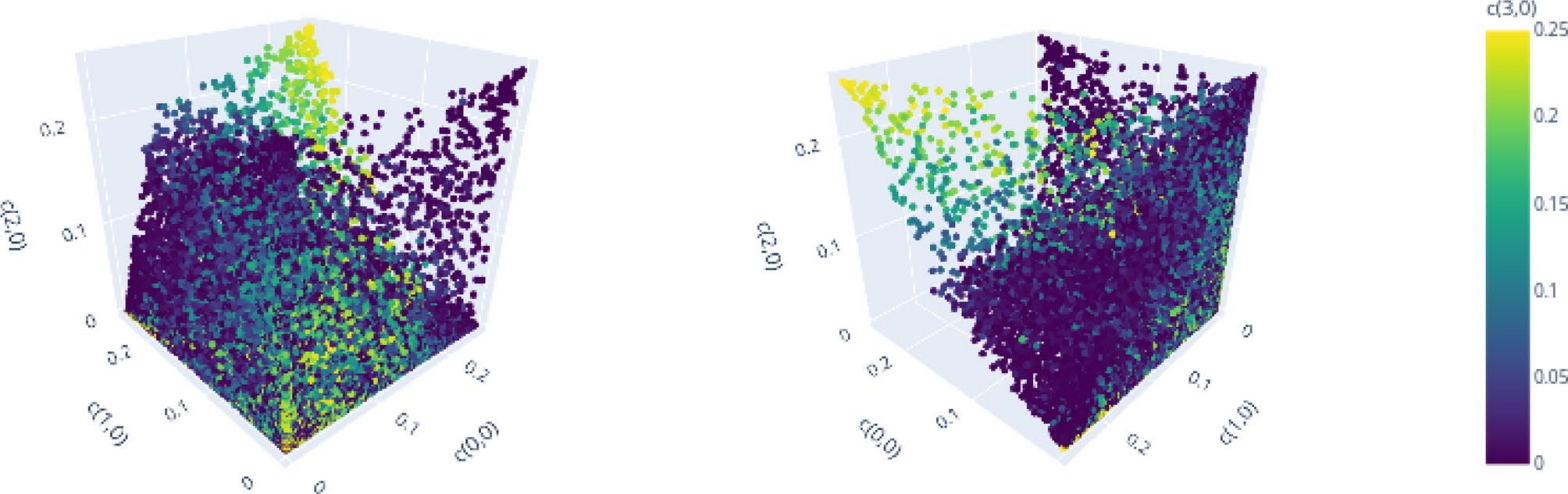
$(c_{0,0}, c_{1,0}, c_{2,0}, c_{3,0})$ for optimized separable states for $d=4$, demonstrating strong correlations of on-line and additionally on-sublattice coordinates. Figure created with Ref.^46^.

**Figure 8:**
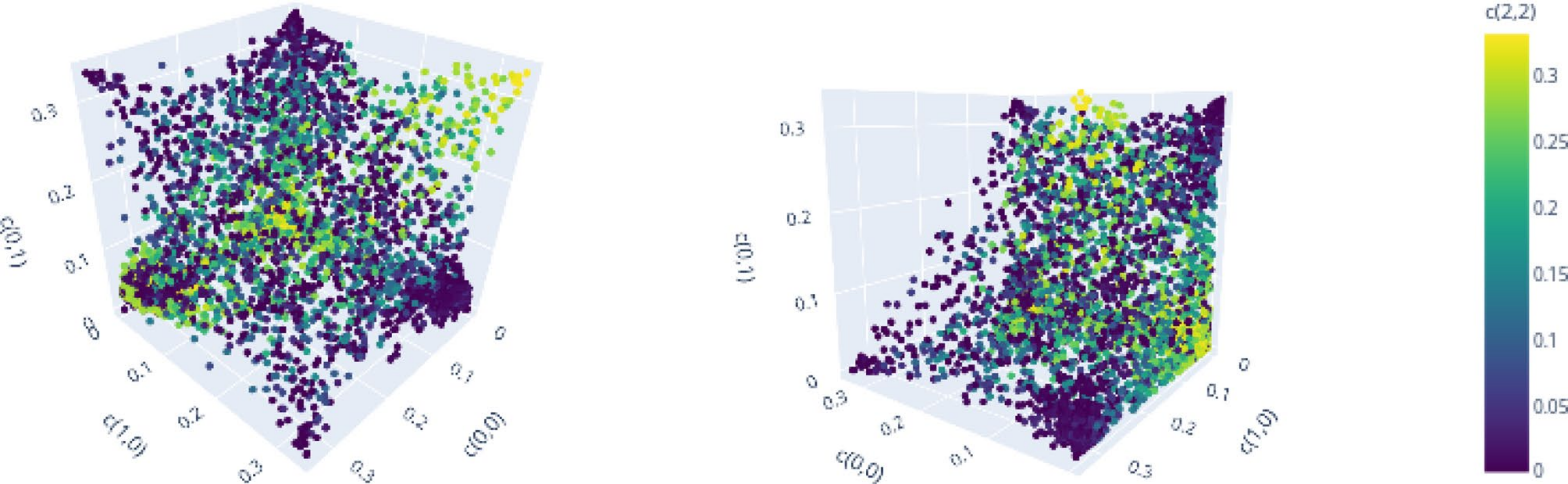
$(c_{0,0}, c_{1,0}, c_{0,1}, c_{2,2})$ for optimized separable states for $d=3$, demonstrating necessity of full-line mixing for separability of states that concentrate most probability on 3 Bell states. Figure created with Ref.^46^.

**Figure 9:**
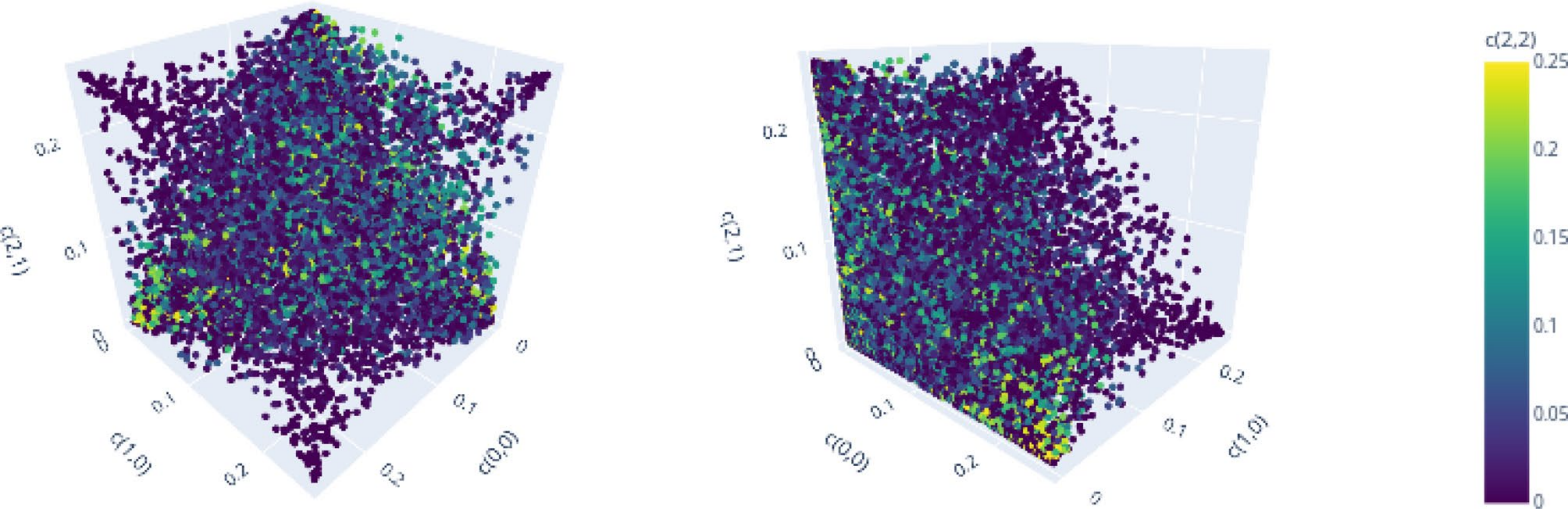
$(c_{0,0}, c_{1,0}, c_{2,1}, c_{2,2})$ for optimized separable states for $d=4$, demonstrating necessity of full-line mixing for separability of states that concentrate most probability on 4 Bell states. Figure created with Ref.^46^.

**Figure 10:**
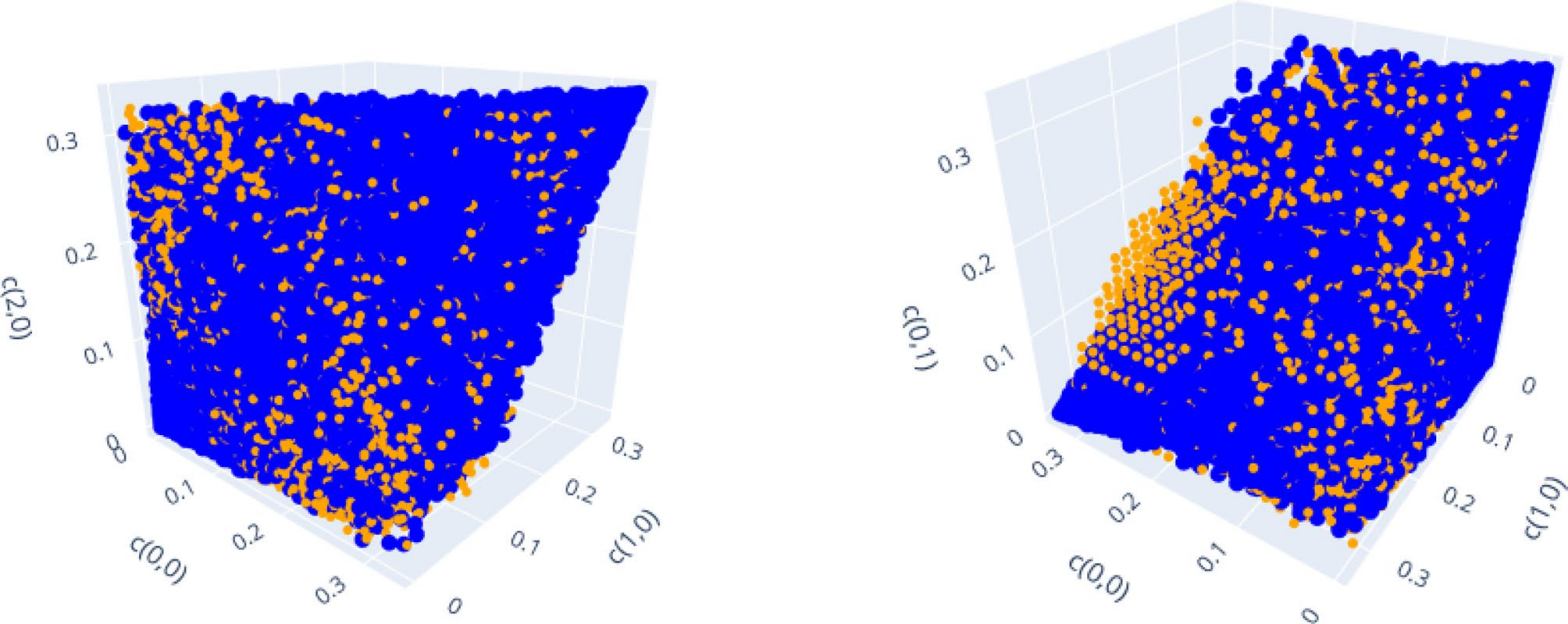
Comparison of projections to on-line (left) vs. off-line (right) coordinates for separable (blue) and bound entangled (orange) states in $d=3$. Off-line projections show weaker geometric restrictions for bound entangled states in certain regions. Figure created with Ref.^46^.

In this section we analyze and compare the structure of the set of separable and PPT entangled Bell diagonal states in dimension $d=3$ and $d=4$. Analyzing the geometric properties of those sets helps to improve geometric methods of entanglement detection or to provide new insights about the geometry of such quantum states in general (see e.g. Ref.^60^). For $d=2$, the geometric properties have been analyzed in detail before^59^. Here, we visualize *d* dimensional projections of PPT states in ${\mathscr {M}}_d$ to demonstrate clear patterns that relate the geometric structure of the set of separable or bound entangled states to the algebraic structure of the Weyl operators and related Bells states. In particular, we make two qualitative observations:

- (Figs. 6, 7, 8 and 9):
  The set of separable states in ${\mathscr {M}}_d$ is geometrically strongly restricted by the *d* element subgroup structure of the Weyl operators. More precisely, the more probability of a separable mixed state is concentrated on *d* Bell states, the closer it needs to be to a subgroup state (for definition see Figs. 1, 2 and related discussion).
- (Fig. 10):
  Similar restrictions related to this subgroup structure are present for bound entangled states, but certain restrictions are stronger for separable states than for bound entangled states. This difference can potentially be leveraged to detect bound entanglement.

The set of separable states in ${\mathscr {M}}_d$ forms a convex geometric body when represented as set of points in $d^2$ dimensional Euclidean space via their coordinates $c_{k,l}$. Naturally, not all coordinates of the $d^2$ dimensional space can be visualized at once, however, the symmetries (see section “Symmetries and their generation”) allow to capture some essential geometric properties, even if only *d* coordinates are shown. The reason for this are *d*-element subgroups that are induced by the underlying ring structure of the Weyl operators (see Figs. 1 and 2). Subgroups can always be mapped onto each other with a corresponding symmetry transformation^19^. Since these transformations conserve the entanglement class and act as permutations on the coordinates $c_{k,l}$, different sets of *d* coordinates will show the same geometric properties if they are symmetric. Here, we consider the special subgroups represented by lines^19,38^ in the discrete phase space that are induced by the consecutive application of simple translations $t_{p,q}$. For example in $d=3$, the indices (*k*, *l*) of the first three coordinates $(c_{0,0}, c_{1,0},c_{2,0})$ form a line (red markers in Fig. 1), because they are related by the translation $t_{1,0}$. The geometric properties of these coordinates are then equivalent to e.g. those of $(c_{0,0},c_{1,1},c_{2,2})$ (blue markers in Fig. 1), as a suitable symmetry transformation relates the collections.

For visualizations we use separable states, which are specifically optimized to be close to the surface of the set of separable states, and random samples of PPT entangled states. We use three coordinates for a 3D-visualization and encode a fourth coordinate by color. Note that all PPT states are contained in the enclosure polytope, so it suffices to limit the range of the coordinates to [0, 1/*d*].

To demonstrate the first observation, we compare the projections of separable states for $d=3$ and $d=4$ to *d* coordinates that relate either all to the same *d*-element subgroup or not. Figure 6 shows the geometric distribution of the first three coordinates on a line and a fourth coordinate encoded by the color for $d=3$ from two point of views. A structure similar to a cone spanned by the corners of the enclosing polytope ${\mathscr {E}}_3$ $\lbrace (1/3,0,0), (0,1/3,0),(0,0,1/3) \rbrace$ and the subgroup state $\lbrace (1/3, 1/3, 1/3) \rbrace$ is visible, while no correlation with the off-line coordinate $c_{0,1}$ can be identified. Note that there are no separable states, for which two coordinates of the line, i.e.. $c_{0,0}$ and $c_{1,0}$, are large, while the remaining line coordinate, i.e.. $c_{2,0}$, is small.

Similar observations can be made for $d=4$, when projecting to the on-line coordinates $(c_{0,0}, c_{1,0}, c_{2,0}, c_{3,0})$ in Fig. 7. Several correlations between the coordinates can be seen. Corresponding to the yellow cone, we see an accumulation of separable states that have high mixing probabilities for all of the four Bell states on the line. The blue cone pointing to the point (1/4, 0, 1/4), on the other hand, relates to a different subgroup with indices $\lbrace (0,0), (2,0), (0,2), (2,2) \rbrace$ (red markers in Fig. 2). Finally, there is no symmetric cone and thus no separable states in the vicinity of (0, 1/4, 1/4). Note that there is also no corresponding subgroup that contains the indices (1, 0) and (2, 0) but excludes the remaining line elements (0, 0) and (3, 0).

The visualizations above demonstrate that for each *d*-element subgroup, there is an accumulation of separable states in the vicinity of the corresponding subgroup state.

Consider now that *d* Bell states share most of the probability of a mixed state. We demonstrate that if those Bell states do not all correspond to the same subgroup, then the mixed state cannot be separable. First, note that if all probability is concentrated on less than *d* states, than at least one probability must exceed 1/*d*, in which case the state cannot be PPT and therefore must be (NPT) entangled (see “Enclosure polytope” on p.4). Below, we visualize projections to coordinates, of which two relate to the same line and remaining coordinates are chosen to be part of different lines. Consider for $d=3$ the projection to the line coordinates defined by $(c_{0,0}, c_{1,0})$ together with the off-line coordinates $c_{0,1}$ and $c_{0,2}$ (Fig. 8). No separable states are present for large values of both on-line coordinates $(c_{0,0}, c_{1,0})$ and large off-line values for $c_{0,1}$ or $c_{2,2}$.

The same observation can be made for $d=4$. Choosing again two coordinates $(c_{0,0}, c_{1,0})$ to define a subgroup and two coordinates that are not part of it, one sees a similar structure in Fig. 9. Again, if the values of the line coordinates $c_{0,0}$ and $c_{1,0}$ are large, there is no separable state for large values of the off-line coordinates $c_{2,1}$ or $c_{2,2}$.

Due to the discussed symmetries, these characterizations hold for all coordinates that have a similar relation regarding their corresponding subgroups. We have therefore demonstrated the first observation: Considering mixed states that concentrate most of the probability on *d* Bell states, similarity to the subgroup states is necessary for separability. Most mixed separable states have their probabilities distributed on all Bell states and are therefore centered around the maximally mixed state. In this case they are likely contained in the convex hull of the subgroup states that define the kernel polytope. We have therefore shown that this linear approximation also captures the relevant geometrical structure of the whole set of separable states in ${\mathscr {M}}_d$. In addition, the observed restrictions allow to construct more effective approximations, which focus on the areas, in which the surface of the set of separable states is curved (see e.g. Fig. 6 (left)) and the linear approximation fails. This can help to improve existing methods of detecting separability (e.g. the extended kernel criterion S1).

We conclude this section by demonstrating the second observation, which states that the geometric restrictions induced by the subgroup structure of the Weyl operators are also present for bound entangled states, but can be distinguished from those for separable states in certain cases.

Consider $d=3$, for which a significant amount of bound entangled states can be classified. In Fig. 10, we again visualize *d* dimensional projections of separable states but also include the detected bound entangled states. Here, we show separable states in blue and bound entangled states in orange. The graphic on the left of Fig. 10 shows the projection to the coordinates $(c_{0,0}, c_{1,0}, c_{2,0})$ that relate to the same subgroup (line). One can see that the projections of the bound entangled states are restricted to the same area as the separable states (also compare Fig. 6 (right)). The described dependence of on-line projections on the related subgroups seems therefore to be a feature of all PPT states.

On the right hand, the visualization of the projection to three coordinates $(c_{0,0}, c_{1,0}, c_{0,1})$, that do not all belong to the same line, shows a relevant difference. The general form of visualized PPT states is still dominated by the convex combination of subgroup states (compare Fig. 8). There are no bound entangled states that concentrate most of the mixing probabilities on three Bell states that do not belong to the same subgroup. Crucially, however, there is a clearly visible region, in which only bound entangled states are present. For given on-line coordinate values $(c_{0,0}, c_{1,0})$, this region is characterized by higher off-line coordinate values $c_{0,1}$ than those accessible for separable projections.

For $d=3$, this demonstrates the second observation: On the one hand, the subgroup structure imposes restrictions on all PPT states that determine the dominant shape of *d* dimensional projections of separable and bound entangled states. On the other hand, there exist *d* dimensional projections under which a subset of bound entangled states is mapped to regions, which do not contain any projected separable states. In principle, knowledge about these regions can be used to construct suitable entanglement witnesses of rank *d* to detect those PPT entangled states in $d^2$ dimensions.

For $d=4$ the number of classified bound entangled states is not large enough to confirm similar characteristics, although none of the classified bound entangled states indicate a qualitative difference to the observations made for $d=3$.

## Discussion and conclusion

In this work we analyzed states of the bipartite system of mixed Bell states, which are related by Weyl transformations, with focus on subsystems with dimension 2, 3 and 4. The entanglement class of these locally maximally mixed states depends on the mixing probabilities and can be separable, NPT/free entangled or for $d > 2$ also PPT/bound entangled.

In order to investigate the properties for $d=4$, i.e. bipartite ququarts, we extended the applied methods recently used to analyze the system of bipartite qutrits^41^. Leveraging a geometric representation, a random sampling of uniformly distributed states can be used to estimate the relative sizes of the entanglement classes via various criteria to detect separability and entanglement, including its bound version. Using this representation together with a group of entanglement class preserving symmetries, related analytical properties and an efficient parameterization^56^ of states allows us to draw several conclusions about the entanglement properties of bipartite qudits and their dependence on the dimension for $d=2,3$ and 4.

A first observation is that the relative number of states with positive partial transposition, so either separable of bound entangled states, decreases very quickly with growing dimension, despite of increasing relative volume of the enclosure polytope ${\mathscr {E}}_d$, known to contain all PPT states. The share of PPT states in the full “magic simplex” ${\mathscr {M}}_d$ decreases from $50\%$ for $d=2$ to $39\%$ for d=3 to $12\%$ for $d=4$. For $d>5$, less than $1\%$ of states are PPT.

The second observation is that significantly less bound entangled states can be detected, while the number of states that cannot be classified is considerably higher for $d=4$ than for $d=3$. To determine the relative volumes of entanglement classes for the enclosure polytope in $d=4$ and compare them to $d=3$, 40000 states in ${\mathscr {E}}_4$ have been created and classified with a probability of success of $96.7 \%$. In principle, the probability of success could further be improved by extension of the numerical analyses and more states could easily be classified. In order to compare to $d=3$, however, the extend of the numerical analysis and the number of states are chosen to result in a similar probability of success and number of PPT states. Limited to the set of PPT states, $77.4 \%$ of states could be successfully differentiated between separable and bound entangled states. The developed methods can be efficiently and repeatedly applied to new unknown states to solve the NP-hard “separability problem” with a probability of success of $77.4 \%$ for Bell diagonal ququarts in the magic simplex. Out of all PPT states in the system ${\mathscr {M}}_4$, at least $75.7\%$ are determined to be separable and $1.7\%$ are classified as bound entangled. The share of detected bound entangled states is clearly smaller than for $d=3$ ($13.9 \%$), however, compared to the results of $d=3$, a higher share (22.6 % vs $5.1\%$) of PPT states could not be classified and it remains unclear whether they are separable or bound entangled.

A third result can be stated regarding the detection capabilities of the applied criteria. The applied detectors for separability (S1, S2) or bound entanglement (E2–E5) are either based on deterministic, analytical conditions (S2, E2, E3, E4) or on a combined collection of numerically generated objects, i.e., vertices for the extension of the kernel polytope for S1 or EW-defining hyperplanes for E5. It can be seen from the large share of unclassified PPT states, as well as from the relative shares of detected states by the criteria in each class, that both types of detectors are less powerful for classification in $d=4$. Out of the analytical criteria for entanglement detection (E2–E4), for $d=4$, only E2 detects a significant amount of bound entangled states, while E3 detects very few and E4 none at all, though it is known that E4 can detect bound entangled Bell diagonal states in $d=4$^53,55^. This is in strong contrast to $d=3$, where the later two criteria allow the detection of $19.1\%$ and $13.5 \%$ of all BOUND classified states. Interestingly, E3 still detects bound entanglement in $d=4$ that cannot be detected with E2. This can also be observed for $d=3$, where E3 can detect more strongly mixed entangled states than E2. The numerical criteria S1 and E5 also show reduced detection capability. Although S1 detects more than $75\%$ of the PPT states as separable, the large number of unclassified states suggests that many separable states might not be detected by the used kernel extension. In addition to the share of classified states, the reduced number of states that are both detected by the analytical criterion E2 and the numerical E5 is also an indication of lower detection power of a single randomly generated EW for $d=4$. another striking difference between $d=3$ and $d=4$ is that S2 does not detect or is very unlikely to detect any separability for $d=4$.

Many BOUND states that are detected by E2 are thus not confirmed by the criterion E5, which clearly shows that the number of generated EWs is not high enough, although more EWs were used than for $d=3$. Two main reasons are likely responsible for the weaker performance in $d=4$: First, the higher dimension of the Euclidean space and second, the different geometric properties of the set of separable states in ${\mathscr {M}}_d$ related to the properties of the Weyl operators and their induced phase space in non-prime dimensions. Both criteria represent approximations of this convex set: S1 represents an polytope approximation from within by identifying separable vertices close to the surface of separable states, while E5 represents an enclosing approximation with the hyperplanes defined by the upper and lower bounds of the EWs. The higher the dimension of the Euclidean space, the more objects (vertices/hyperplanes) are needed for a sufficient approximation and a generated set of objects may not be sufficient to achieve a comparable probability of success. The geometric properties of the (unknown) convex body formed by separable states are also relevant, as they determine the results of optimization procedures over the set of separable states in the whole Hilbert space, on which the generation of EWs and separable vertices rely.

Finally, we used classified separable and bound entangled states to enable visual analyses concerning the structure of PPT states in ${\mathscr {M}}_d$. We argued that relevant information can be extracted by considering projections to *d* coordinates, due to the special symmetries in ${\mathscr {M}}_d$ for $d=3$ and $d=4$. Two main qualitative observations were made that relate the structure of separable and bound entangled states to the algebraic subgroup structure of the Weyl operators.

On the one hand, it was shown that the states defined by the induced subgroup structure of the Weyl operators, which are used to define the kernel polytope ${\mathscr {K}}_d$, also determine the dominant geometric shape of the body of separable states. It was demonstrated for both $d=3$ and $d=4$ that a mixed state that concentrates most of the probability on *d* Bell states is separable, only if all Bell states relate to the same subgroup. This insights can be used to construct better approximations of the set of separable states and thus to improve methods to detect separability among PPT states.

On the other hand, we have demonstrated that similar restrictions related to the subgroups hold as well for the *d* dimensional projections of bound entangled states. Importantly, however, for some projections, these restrictions seem to be less strict for bound entangled states than those imposed on separable states. As a consequence, there are *d* dimensional areas, which are not reachable for projections of separable states. In principle, PPT states that are projected to these areas can be classified as entangled by geometric criteria that are defined in *d* dimensional space, instead of the full $(d^2-1)$ dimensional Hilbert space.

In conclusion, the methods for state generation and entanglement analysis applied to the system of $d=3$ can also be successfully applied to $d=4$ (with extensions), although with reduced effectiveness. Nonetheless, the presented methods solve the separability problem for ${\mathscr {M}}_4$ to a large extend, since any unknown state in ${\mathscr {M}}_4$ can efficiently be classified as separable or (bound) entangled with high probability of success. Significant differences in the relative volumes of the entanglement classes and in the detection capabilities of criteria for separability and entanglement are observed for ${\mathscr {M}}_3$ and ${\mathscr {M}}_4$. Relating the algebraic structure of the Weyl operators to the geometry of ${\mathscr {M}}_d$, qualitative observations could be made that characterize the structure of PPT states and propose new potential criteria to detect bound entanglement in ${\mathscr {M}}_d$. These contributions can serve as starting point to further improve the methods for classification and the general understanding of the entanglement structure of Bell diagonal qudits or general quantum states. On the one hand, further numerical investigations, extending the current results in terms of higher dimensions $d \ge 5$ or the structure of bound entangled quantum states, are possible. The applied sampling methods remain efficient for Bell diagonal states and thus allow the confirmation of results concerning the exponential decrease of volume for separable general quantum states with growing dimension as reported in Ref.^42,43^ or the observation of special properties for Bell diagonal states. Recently, a sequentially constrained Monte Carlo sampler (SCMCS) for quantum states was proposed^61^, which allows efficient sampling of quantum states subject to constraints like PPT or detection properties for specific entanglement criteria. This method could be used to generate general bound entangled quantum states and compare their properties to those of Bell diagonal states or for the detailed investigation of detection capabilities of certain entanglement witnesses. On the other hand, the reported structures of separable and bound entangled Bell diagonal states in ${\mathscr {M}}_d$ indicate properties that can be related to those of the Weyl operators and their induced phase space structure. The presented methods help to create, confirm or refute hypothesis about the structure of separable or entangled Bell diagonal states for different dimensions. Thus, they could provide a new accesses to the separability problem, the detection of bound entanglement or application relevant properties of quantum systems using entangled qudits.

## Supplementary Information

Supplementary Information.

## Data Availability

All analyzed datasets were generated during the current study and are available from the corresponding author on reasonable request. The software used to generate the reported results is published as registered open source package “BellDiagonalQudits.jl”^62^ available at https://github.com/kungfugo/BellDiagonalQudits.jl.

## References

[1] Cirac JI, Ekert AK, Huelga SF, Macchiavello C (1999). Distributed quantum computation over noisy channels. Phys. Rev. A.

[2] Ekert AK (1991). Quantum cryptography based on bell’s theorem. Phys. Rev. Lett..

[3] Georgescu IM, Ashhab S, Nori F (2014). Quantum simulation. Rev. Mod. Phys..

[4] Deng F-G, Long GL, Liu X-S (2003). Two-step quantum direct communication protocol using the Einstein–Podolsky–Rosen pair block. Phys. Rev. A.

[5] Sheng Y-B, Zhou L, Long G-L (2022). One-step quantum secure direct communication. Sci. Bull..

[6] Nielsen, M. A. & Chuang, I. L. *Quantum Computation and Quantum Information* (Cambridge University Press, 2000).

[7] Einstein A, Podolsky B, Rosen N (1935). Can quantum-mechanical description of physical reality be considered complete?. Phys. Rev..

[8] Bell JS (1964). On the Einstein Podolsky Rosen paradox. Physics Physique Fizika.

[9] Hensen B (2015). Loophole-free bell inequality violation using electron spins separated by 1.3 kilometres. Nature.

[10] Moskal P (2016). Time resolution of the plastic scintillator strips with matrix photomultiplier readout for j-PET tomograph. Phys. Med. Biol..

[11] Moskal P, Stepien E (2020). Prospects and clinical perspectives of total-body pet imaging using plastic scintillators. PET Clin..

[12] Hiesmayr BC, Moskal P (2017). Genuine multipartite entanglement in the 3-photon decay of positronium. Sci. Rep..

[13] Hiesmayr BC, Moskal P (2019). Witnessing entanglement in compton scattering processes via mutually unbiased bases. Sci. Rep..

[14] Cozzolino D, Da Lio B, Bacco D, Oxenløwe LK (2019). High-dimensional quantum communication: Benefits, progress, and future challenges. Adv. Quantum Technol..

[15] Wang Y, Hu Z, Sanders BC, Kais S (2020). Qudits and high-dimensional quantum computing. Front. Phys..

[16] Braunstein SL, Mann A, Revzen M (1992). Maximal violation of bell inequalities for mixed states. Phys. Rev. Lett..

[17] Sych D, Leuchs G (2009). A complete basis of generalized bell states. New J. Phys..

[18] Bennett CH (1993). Teleporting an unknown quantum state via dual classical and Einstein–Podolsky–Rosen channels. Phys. Rev. Lett..

[19] Baumgartner B, Hiesmayr BC, Narnhofer H (2007). A special simplex in the state space for entangled qudits. J. Phys. A.

[20] Peres A (1996). Separability criterion for density matrices. Phys. Rev. Lett..

[21] Horodecki M, Horodecki P, Horodecki R (1996). Separability of mixed states: Necessary and sufficient conditions. Phys. Lett. A.

[22] Horodecki M, Horodecki P, Horodecki R (1998). Mixed-state entanglement and distillation: Is there a bound entanglement in nature?. Phys. Rev. Lett..

[23] Bej P, Halder S (2021). Unextendible product bases, bound entangled states, and the range criterion. Phys. Lett. A.

[24] Lockhart J, Gühne O, Severini S (2018). Entanglement properties of quantum grid states. Phys. Rev. A.

[25] Bruß D, Peres A (2000). Construction of quantum states with bound entanglement. Phys. Rev. A.

[26] Slater, P. B. Jagged islands of bound entanglement and witness-parameterized probabilities. *arXiv Quantum Physics* ( 2019). https://doi.org/10.48550/arXiv.1905.09228.

[27] Choi M-D (1980). Some assorted inequalities for positive linear maps on c*-algebras. J. Oper. Theory.

[28] Chruściński D, Sarbicki G (2014). Entanglement witnesses: Construction, analysis and classification. J. Phys. A.

[29] Kalev A, Bae J (2013). Optimal approximate transpose map via quantum designs and its applications to entanglement detection. Phys. Rev. A.

[30] Bae J (2017). Designing quantum information processing via structural physical approximation. Rep. Prog. Phys..

[31] Korbicz JK, Almeida ML, Bae J, Lewenstein M, Acín A (2008). Structural approximations to positive maps and entanglement-breaking channels. Phys. Rev. A.

[32] Huber M, Mintert F, Gabriel A, Hiesmayr BC (2010). Detection of high-dimensional genuine multipartite entanglement of mixed states. Phys. Rev. Lett..

[33] Augusiak R, Bae J, Tura Brugués J, Lewenstein M (2014). Checking the optimality of entanglement witnesses: An application to structural physical approximations. J. Phys. A: Math. Theor..

[34] Hiesmayr BC, Löffler W (2013). Complementarity reveals bound entanglement of two twisted photons. New J. Phys..

[35] Gurvits, L. Classical deterministic complexity of Edmonds’ problem and quantum entanglement. In *Proceedings of the Thirty-Fifth Annual ACM Symposium on Theory of Computing, STOC ’03* 10-19 (Association for Computing Machinery, 2003). 10.1145/780542.780545.

[36] Gharibian S (2008). Strong np-hardness of the quantum separability problem. Quantum Inf. Comput..

[37] Werner RF (2001). All teleportation and dense coding schemes. J. Phys. A.

[38] Baumgartner B, Hiesmayr BC, Narnhofer H (2006). State space for two qutrits has a phase space structure in its core. Phys. Rev. A.

[39] Terhal BM (2000). Bell inequalities and the separability criterion. Phys. Lett. A.

[40] Bae J, Chruściński D, Hiesmayr BC (2020). Mirrored entanglement witnesses. npj Quantum Inf..

[41] Popp C, Hiesmayr BC (2022). Almost complete solution for the np-hard separability problem of bell diagonal qutrits. Sci. Rep..

[42] Życzkowski K, Horodecki P, Sanpera A, Lewenstein M (1998). Volume of the set of separable states. Phys. Rev. A.

[43] Zyczkowski K (1999). Volume of the set of separable states. II. Phys. Rev. A.

[44] Hill SA, Wootters WK (1997). Entanglement of a pair of quantum bits. Phys. Rev. Lett..

[45] Baumgartner B, Hiesmayr BC, Narnhofer H (2008). The geometry of bipartite qutrits including bound entanglement. Phys. Lett. A.

[46] Plotly graphing libraries (v0.18.6). https://plotly.com/julia/.

[47] Bae J (2009). Detection and typicality of bound entangled states. Phys. Rev. A.

[48] Willms AR (2021). Uniform Sampling on the Standard Simplex. Mo. J. Math. Sci..

[49] Chen K, Wu L-A (2002). A matrix realignment method for recognizing entanglement. Quantum Inf. Comput..

[50] Wootters WK (1998). Entanglement of formation of an arbitrary state of two qubits. Phys. Rev. Lett..

[51] Wootters WK, Fields BD (1989). Optimal state-determination by mutually unbiased measurements. Ann. Phys..

[52] Bandyopadhyay S, Boykin PO, Roychowdhury VP, Vatan F (2002). A new proof for the existence of mutually unbiased bases. Algorithmica.

[53] Bae, J., Bera, A., Chruściński, D., Hiesmayr, B. C. & McNulty, D. How many measurements are needed to detect bound entangled states? (2021).

[54] Spengler C, Huber M, Brierley S, Adaktylos T, Hiesmayr BC (2012). Entanglement detection via mutually unbiased bases. Phys. Rev. A.

[55] Hiesmayr BC (2021). Detecting entanglement can be more effective with inequivalent mutually unbiased bases. New J. Phys..

[56] Spengler C, Huber M, Hiesmayr BC (2010). A composite parameterization of unitary groups, density matrices and subspaces. J. Phys. A: Math. Theor.

[57] Forets M, Schilling C (2021). Lazysets.jl: Scalable symbolic-numeric set computations$$^*$$. Proc. JuliaCon Conf..

[58] Chruściński D, Pittenger AO (2008). Generalized circulant densities and a sufficient condition for separability. J. Phys. A: Math. Theor..

[59] Bertlmann RA, Narnhofer H, Thirring W (2002). Geometric picture of entanglement and bell inequalities. Phys. Rev. A.

[60] Bengtsson, I. & Życzkowski, K. *Geometry of Quantum States: An Introduction to Quantum Entanglement* (Cambridge University Press, 2006).

[61] Li, W., Han, R., Shang, J., Ng, H. K. & Englert, B.-G. *Sequentially Constrained Monte Carlo Sampler for Quantum States*. arXiv:org/abs/2109.14215 (2021).

[62] Popp C (2023). BellDiagonalQudits: A package for entanglement analyses of mixed maximally entangled qudits. J. Open Source Softw..

