# Supplementary material for: Comparing bound entanglement of bell diagonal pairs of qutrits and ququarts
Source: Sci Rep. 2023 Feb 4;13:2037. doi: 10.1038/s41598-023-29211-w (PMC9899246; doi:10.1038/s41598-023-29211-w)
Supplement: Supplementary file 1 — Supplementary Information. [file 41598_2023_29211_MOESM1_ESM.pdf]

Appendix

A1: MUB sets for $d=3$ and $d=4$

The following sets of bases are known to be mutually unbiased[47] and non-decomposable, i.e. able to detect bound entanglement[46]. Note, that other MUBs exist, which generally detect different sets of entangled states[46,48]. For $d=3$ and $d=4$ and $w=e^{\frac{2\pi i}{d}}$, the used bases with basis vectors combined to matrix columns and represented in the computational basis read as follows:

d = 3:

$$B_1=\begin{pmatrix}1&0&0\\0&1&0\\0&0&1\end{pmatrix},\ B_2=\frac{1}{\sqrt{3}}\begin{pmatrix}1&1&1\\1&w&w^2\\1&w^2&w\end{pmatrix},\ B_3=\frac{1}{\sqrt{3}}\begin{pmatrix}1&1&1\\w&w^2&1\\w&1&w^2\end{pmatrix},\ B_4=\frac{1}{\sqrt{3}}\begin{pmatrix}1&1&1\\w^2&1&w\\w^2&w&1\end{pmatrix}$$

d = 4:

$$B_1=\begin{pmatrix}1&0&0&0\\0&1&0&0\\0&0&1&0\\0&0&0&1\end{pmatrix},\ B_2=\frac{1}{2}\begin{pmatrix}1&1&1&1\\1&1&-1&-1\\1&-1&-1&1\\1&-1&1&-1\end{pmatrix},\ B_3=\frac{1}{2}\begin{pmatrix}1&1&1&1\\-i&-i&i&i\\-i&i&i&-i\\-1&1&-1&1\end{pmatrix},$$

$$B_4=\frac{1}{2}\begin{pmatrix}1&1&1&1\\-1&-1&1&1\\-i&i&i&-i\\-i&i&-i&i\end{pmatrix},\ B_5=\frac{1}{2}\begin{pmatrix}1&1&1&1\\-i&-i&i&i\\-1&1&1&-1\\-i&i&-i&i\end{pmatrix}$$

References

1. Nielsen, M. A. & Chuang, I. L. *Quantum Computation and Quantum Information* (Cambridge University Press, 2000).
2. Einstein, A., Podolsky, B. & Rosen, N. Can quantum-mechanical description of physical reality be considered complete? *Phys. Rev.* **47**, 777–780 (1935). URL https://link.aps.org/doi/10.1103/PhysRev.47.777.
3. Bell, J. S. On the einstein podolsky rosen paradox. *Physics Physique Fizika* **1**, 195–200 (1964). URL https://link.aps.org/doi/10.1103/PhysicsPhysiqueFizika.1.195.
4. Hensen, B. *et al.* Loophole-free bell inequality violation using electron spins separated by 1.3 kilometres. *Nature* **526** (2015).
5. Moskal, P. *et al.* Time resolution of the plastic scintillator strips with matrix photomultiplier readout for j-PET tomograph. *Physics in Medicine and Biology* **61**, 2025–2047 (2016). URL https://doi.org/10.1088/0031-9155/61/5/2025.
6. Moskal, P. & Stepien, E. Prospects and clinical perspectives of total-body pet imaging using plastic scintillators. *PET Clinics* **15** (2020). URL https://doi.org/10.1016/j.cpet.2020.06.009.
7. Hiesmayr, B. C. & Moskal, P. Genuine multipartite entanglement in the 3-photon decay of positronium. *Scientific Reports* **7**, 15349 (2017). URL https://doi.org/10.1038/s41598-017-15356-y.
8. Hiesmayr, B. C. & Moskal, P. Witnessing entanglement in compton scattering processes via mutually unbiased bases. *Scientific Reports* **9**, 8166 (2019). URL https://doi.org/10.1038/s41598-019-44570-z.
9. Cozzolino, D., Da Lio, B., Bacco, D. & Oxenløwe, L. K. High-dimensional quantum communication: Benefits, progress, and future challenges. *Advanced Quantum Technologies* **2**, 1900038 (2019). URL https://doi.org/10.1002/qute.201900038.
10. Wang, Y., Hu, Z., Sanders, B. C. & Kais, S. Qudits and high-dimensional quantum computing. *Frontiers in Physics* **8**, 479 (2020). URL https://www.frontiersin.org/article/10.3389/fphy.2020.589504.
11. Braunstein, S. L., Mann, A. & Revzen, M. Maximal violation of bell inequalities for mixed states. *Phys. Rev. Lett.* **68**, 3259–3261 (1992). URL https://link.aps.org/doi/10.1103/PhysRevLett.68.3259.
12. Sych, D. & Leuchs, G. A complete basis of generalized bell states. *New Journal of Physics* **11**, 013006 (2009). URL https://doi.org/10.1088/1367-2630/11/1/013006.
13. Bennett, C. H. *et al.* Teleporting an unknown quantum state via dual classical and einstein-podolsky-rosen channels. *Phys. Rev. Lett.* **70**, 1895–1899 (1993). URL https://link.aps.org/doi/10.1103/PhysRevLett.70.1895.
14. Baumgartner, B., Hiesmayr, B. C. & Narnhofer, H. A special simplex in the state space for entangled qudits. *J. Phys. A: Math. Theor.* **40**, 7919 (2007). URL https://doi.org/10.1088/1751-8113/40/28/S03.
15. Peres, A. Separability criterion for density matrices. *Phys. Rev. Lett.* **77**, 1413–1415 (1996). URL https://link.aps.org/doi/10.1103/PhysRevLett.77.1413.
16. Horodecki, M., Horodecki, P. & Horodecki, R. Separability of mixed states: necessary and sufficient conditions. *Physics Letters A* **223**, 1–8 (1996). URL https://www.sciencedirect.com/science/article/pii/S0375960196007062.
17. Horodecki, M., Horodecki, P. & Horodecki, R. Mixed-state entanglement and distillation: Is there a “bound” entanglement in nature? *Phys. Rev. Lett.* **80**, 5239–5242 (1998). URL https://link.aps.org/doi/10.1103/PhysRevLett.80.5239.
18. Bej, P. & Halder, S. Unextendible product bases, bound entangled states, and the range criterion. *Physics Letters A* **386**, 126992 (2021). URL https://www.sciencedirect.com/science/article/pii/S0375960120308598.
19. Lockhart, J., Gühne, O. & Severini, S. Entanglement properties of quantum grid states. *Phys. Rev. A* **97**, 062340 (2018). URL https://link.aps.org/doi/10.1103/PhysRevA.97.062340.
20. Bruß, D. & Peres, A. Construction of quantum states with bound entanglement. *Phys. Rev. A* **61**, 030301 (2000). URL https://link.aps.org/doi/10.1103/PhysRevA.61.030301.
21. Slater, P. B. Jagged islands of bound entanglement and witness-parameterized probabilities. *arXiv: Quantum Physics* (2019). URL https://doi.org/10.48550/arXiv.1905.09228.
22. Choi, M.-D. Some assorted inequalities for positive linear maps on c*-algebras. *Journal of Operator Theory* **4** (1980).

23. Chruściński, D. & Sarbicki, G. Entanglement witnesses: construction, analysis and classification. *J. Phys. A: Math. Theor.* **47**, 483001 (2014). URL https://doi.org/10.1088/1751-8113/47/48/483001.

24. Kalev, A. & Bae, J. Optimal approximate transpose map via quantum designs and its applications to entanglement detection. *Phys. Rev. A* **87**, 062314 (2013). URL https://link.aps.org/doi/10.1103/PhysRevA.87.062314.

25. Bae, J. Designing quantum information processing via structural physical approximation. *Reports on Progress in Physics* **80**, 104001 (2017). URL https://doi.org/10.1088/1361-6633/aa7d45.

26. Korbicz, J. K., Almeida, M. L., Bae, J., Lewenstein, M. & Acín, A. Structural approximations to positive maps and entanglement-breaking channels. *Phys. Rev. A* **78**, 062105 (2008). URL https://link.aps.org/doi/10.1103/PhysRevA.78.062105.

27. Huber, M., Mintert, F., Gabriel, A. & Hiesmayr, B. C. Detection of high-dimensional genuine multipartite entanglement of mixed states. *Phys. Rev. Lett.* **104**, 210501 (2010). URL https://link.aps.org/doi/10.1103/PhysRevLett.104.210501.

28. Augusiak, R., Bae, J., Tura Brugués, J. & Lewenstein, M. Checking the optimality of entanglement witnesses: An application to structural physical approximations. *J. Phys. A: Math. Theor.* **47** (2014). URL https://iopscience.iop.org/article/10.1088/1751-8113/47/6/065301.

29. Hiesmayr, B. C. & Löffler, W. Complementarity reveals bound entanglement of two twisted photons. *New J. Phys* **15**, 083036 (2013). URL https://doi.org/10.1088/1367-2630/15/8/083036.

30. Gurvits, L. Classical deterministic complexity of edmonds' problem and quantum entanglement. In *Proceedings of the Thirty-Fifth Annual ACM Symposium on Theory of Computing*, STOC '03, 10–19 (Association for Computing Machinery, New York, NY, USA, 2003). URL https://doi.org/10.1145/780542.780545.

31. Werner, R. F. All teleportation and dense coding schemes. *Journal of Physics A: Mathematical and General* **34**, 7081–7094 (2001). URL https://doi.org/10.1088/0305-4470/34/35/332.

32. Baumgartner, B., Hiesmayr, B. C. & Narnhofer, H. State space for two qutrits has a phase space structure in its core. *Phys. Rev. A* **74**, 032327 (2006). URL https://link.aps.org/doi/10.1103/PhysRevA.74.032327.

33. Terhal, B. M. Bell inequalities and the separability criterion. *Physics Letters A* **271**, 319–326 (2000). URL https://www.sciencedirect.com/science/article/pii/S0375960100004011.

34. Bae, J., Chruściński, D. & Hiesmayr, B. C. Mirrored entanglement witnesses. *npj Quantum Information* **6** (2020). URL https://doi.org/10.1038/s41534-020-0242-z.

35. Popp, C. & Hiesmayr, B. C. Almost complete solution for the np-hard separability problem of bell diagonal qutrits. *Scientific Reports* **12**, 12472 (2022). URL https://doi.org/10.1038/s41598-022-16225-z.

36. Życzkowski, K., Horodecki, P., Sanpera, A. & Lewenstein, M. Volume of the set of separable states. *Phys. Rev. A* **58**, 883–892 (1998). URL https://link.aps.org/doi/10.1103/PhysRevA.58.883.

37. Zyczkowski, K. Volume of the set of separable states. ii. *Phys. Rev. A* **60**, 3496–3507 (1999). URL https://link.aps.org/doi/10.1103/PhysRevA.60.3496.

38. Hill, S. A. & Wootters, W. K. Entanglement of a pair of quantum bits. *Phys. Rev. Lett.* **78**, 5022–5025 (1997). URL https://link.aps.org/doi/10.1103/PhysRevLett.78.5022.

39. Baumgartner, B., Hiesmayr, B. C. & Narnhofer, H. The geometry of bipartite qutrits including bound entanglement. *Physics Letters A* **372**, 2190–2195 (2008). URL https://www.sciencedirect.com/science/article/pii/S0375960107016507.

40. Bae, J. *et al.* Detection and typicality of bound entangled states. *Phys. Rev. A* **80**, 022317 (2009). URL https://link.aps.org/doi/10.1103/PhysRevA.80.022317.

41. Willms, A. R. Uniform Sampling on the Standard Simplex. *Missouri Journal of Mathematical Sciences* **33**, 119–124 (2021). URL https://doi.org/10.35834/2021/3301119.

42. Chen, K. & Wu, L.-A. A matrix realignment method for recognizing entanglement. *Quantum Information and Computation* **3** (2002). URL https://doi.org/10.48550/arXiv.quant-ph/0205017.

43. Wootters, W. K. Entanglement of formation of an arbitrary state of two qubits. *Phys. Rev. Lett.* **80**, 2245–2248 (1998). URL https://link.aps.org/doi/10.1103/PhysRevLett.80.2245.

44. Wootters, W. K. & Fields, B. D. Optimal state-determination by mutually unbiased measurements. *Annals of Physics* **191**, 363–381 (1989). URL https://www.sciencedirect.com/science/article/pii/0003491689903229.

45. Bandyopadhyay, S., Boykin, P. O., Roychowdhury, V. P. & Vatan, F. A new proof for the existence of mutually unbiased bases. *Algorithmica* **34**, 512–528 (2002). URL https://doi.org/10.1007/s00453-002-0980-7.

46. Bae, J., Bera, A., Chruściński, D., Hiesmayr, B. C. & McNulty, D. How many measurements are needed to detect bound entangled states? (2021). 2108.01109.

47. Spengler, C., Huber, M., Brierley, S., Adaktylos, T. & Hiesmayr, B. C. Entanglement detection via mutually unbiased bases. *Phys. Rev. A* **86**, 022311 (2012). URL https://link.aps.org/doi/10.1103/PhysRevA.86.022311.

48. Hiesmayr, B. C. *et al.* Detecting entanglement can be more effective with inequivalent mutually unbiased bases. *New Journal of Physics* **23**, 093018 (2021). URL https://doi.org/10.1088/1367-2630/ac20ea.

49. Spengler, C., Huber, M. & Hiesmayr, B. C. A composite parameterization of unitary groups, density matrices and subspaces. *J. Phys. A: Math. Theor* **43**, 385306 (2010). URL https://doi.org/10.1088/1751-8113/43/38/385306.

50. Forets, M. & Schilling, C. Lazysets.jl: Scalable symbolic-numeric set computations*. *Proceedings of the JuliaCon Conferences* **1**, 97 (2021). URL https://doi.org/10.21105/jcon.00097.

51. Chruściński, D. & Pittenger, A. O. Generalized circulant densities and a sufficient condition for separability. *J. Phys. A: Math. Theor.* **41**, 385301 (2008). URL https://doi.org/10.1088/1751-8113/41/38/385301.

52. Bertlmann, R. A., Narnhofer, H. & Thirring, W. Geometric picture of entanglement and bell inequalities. *Phys. Rev. A* **66**, 032319 (2002). URL https://link.aps.org/doi/10.1103/PhysRevA.66.032319.

53. Bengtsson, I. & Życzkowski, K. *Geometry of Quantum States: An Introduction to Quantum Entanglement* (Cambridge University Press, 2006).

54. Li, W., Han, R., Shang, J., Ng, H. K. & Englert, B.-G. Sequentially constrained monte carlo sampler for quantum states (2021). URL https://arxiv.org/abs/2109.14215.
